# Learning from virtual experiments to assist users of Small Angle Neutron Scattering in model selection

**DOI:** 10.1038/s41598-024-65712-y

**Published:** 2024-07-01

**Authors:** José Ignacio Robledo, Henrich Frielinghaus, Peter Willendrup, Klaus Lieutenant

**Affiliations:** 1 Jülich Centre for Neutron Science 2 (JCNS2), Forschungszentrum Jülich, 52428 Jülich, Germany; 2 Jülich Centre for Neutron Science 4 (JCNS4), Forschungszentrum Jülich, 85748 Garching, Germany; 3 Physics Department, Technical University of Denmark, 2800 Kongens Lyngby, Denmark; 4 Data Management and Software Centre (DMSC), European Spallation Source, 2800 Kongens Lyngby, Denmark

**Keywords:** Machine learning, Neutron, Convolutional Neural Network, SANS dataset, Monte Carlo, Materials science, Techniques and instrumentation

## Abstract

In this work, we combine the advantages of virtual Small Angle Neutron Scattering (SANS) experiments carried out by Monte Carlo simulations with the recent advances in computer vision to generate a tool that can assist SANS users in small angle scattering model selection. We generate a dataset of almost 260.000 SANS virtual experiments of the SANS beamline KWS-1 at FRM-II, Germany, intended for Machine Learning purposes. Then, we train a recommendation system based on an ensemble of Convolutional Neural Networks to predict the SANS model from the two-dimensional scattering pattern measured at the position-sensitive detector of the beamline. The results show that the CNNs can learn the model prediction task, and that this recommendation system has a high accuracy in the classification task on 46 different SANS models. We also test the network with real data and explore the outcome. Finally, we discuss the reach of counting with the set of virtual experimental data presented here, and of such a recommendation system in the SANS user data analysis procedure.

## Introduction

Monte Carlo simulations have proven to be fundamental in revolutionizing the design and optimization of neutron instruments of large-scale facilities^1–4^. It is clear that the precision and reliability of experimental outcomes greatly depend on the design and configuration of the neutron instrument, but constructing and fine-tuning real-world instruments at a large-scale facility can be time-consuming and expensive. The use of Monte Carlo (MC) simulations provides researchers with a cost-effective and efficient virtual testing ground for exploring various instrument setups, optimizing parameters, and identifying the most promising configurations for specific experiments.

Small Angle Neutron Scattering^5^ (SANS) experiments provide valuable insights into the structural and morphological properties of materials at the nanoscale^6–8^ making SANS an important method to investigate nanometer-sized biological systems, polymers, magnetic particles, etc. While an averaged particle size is easily available from SANS data, detailed information about size distribution and geometry are difficult to obtain, making any support by Machine Learning (ML) algorithms desirable. Some efforts in using ML algorithms for SANS data have already been made in one-dimensional curves^9–11^ and in two-dimensional images^12^ showing great potential on small datasets. Nevertheless, algorithms like deep neural networks require large datasets and this becomes a problem in neutron science because conducting experiments in real-world settings is time-consuming (due to complex sample preparations, experimental setups, and data collection processes). Moreover, most of the available data lack metadata and have format compatibility issues that do not allow easy usability. As a consequence, there is only a limited amount of experimental data, which hinders the construction of a comprehensive and large-scale dataset required for machine learning applications.

In the field of SANS, the integration of MC simulations for generating datasets presents an interesting solution to overcome the inherent time constraints and challenges associated with data acquisition. The use of MC simulations allows researchers to virtually explore an extensive range of the parameter space of the simulation and sample configurations efficiently. By generating synthetic data, these simulations enable the construction of sizable and structured datasets that can be utilized for training ML algorithms. In addition to efficiently generating datasets for ML applications, MC simulations of SANS experiments offer an advantage by providing the ground truth for the analytical model of the sample description. This feature enables the use of supervised learning algorithms, which need labeled data for training. These algorithms can be applied effectively, learning from the simulations’ ground truth and extrapolating this knowledge to analyze real experimental data with remarkable accuracy. The synergy between MC simulations and supervised learning holds the potential to revolutionize the analysis of neutron scattering data, offering researchers a robust and efficient tool-set for gaining deeper insights into complex materials. By employing parallelization techniques, we can harness the full potential of modern computing hardware, drastically reducing simulation time and expediting the generation of large datasets.

In this work we describe the generation of a dataset of 260.000 SANS virtual experiments of the KWS-1 beamline^13^ at the FRM-II reactor, in Germany, for machine learning purposes. This dataset is intended to be open to the community. We then show a machine learning approach example using this dataset by creating a recommendation system of SANS models from 2-dimensional (2D) images generated by the Position Sensitive Detector (PSD) of this beamline, which is based on convolutional neural networks (CNNs). Finally, we evaluate and discuss the performance of our model with real experimental data.

## Results

### SANS dataset of KWS-1 virtual experiments

**Figure 1:**
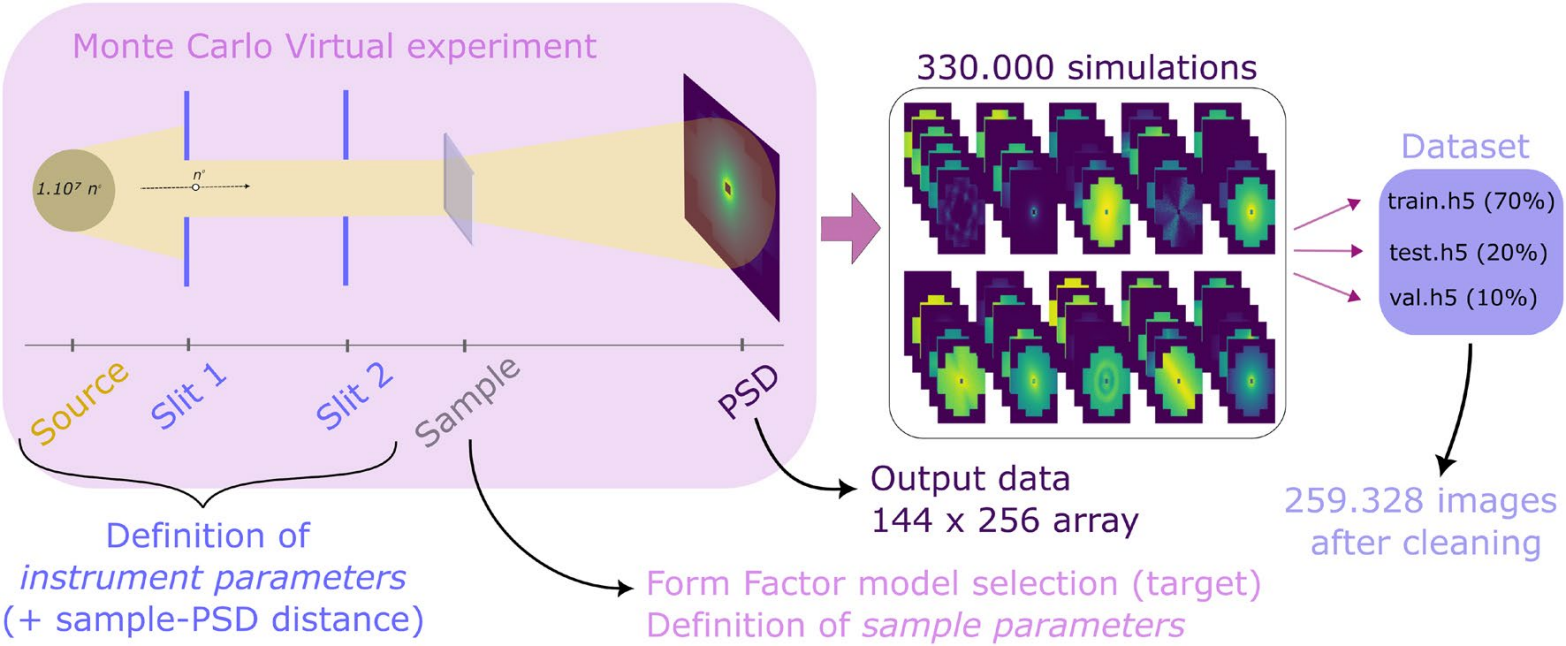
Schematic of how the database was generated. The virtual experiment arrangement is shown on the upper left, and consists on a virtual neutron source, two slits that define the divergence of the incident beam, a sample that scatters the incident beam, and a position sensitive detector (PSD). A beamstop was also used but is not shown in the figure. A set of 330.000 simulations of our virtual experiment setup were simulated under different instrument and sample parameter configurations. After cleaning the database of null images and low intensity images, the resulting 259.328 data arrays are separated in train, test, and validation partitions, which are stored in HDF files (.h5).

We generated a dataset of 259.328 virtual experiments using McStas^2,3^. In each simulation, we varied the sample description and the KWS-1 instrument setting systematically (see Methods section for details) to explore the small angle scattering pattern variability on a two-dimensional position sensitive detector. In Fig. 1 we show a schematic of the experimental setup from the MC source until the position sensitive detector (PSD). All simulations were performed for $N=10^7$ neutrons leaving the source, therefore MC simulations were accelerated using the multi-threading capabilities of the SANS components available in McStas. Each element of the dataset is the scattering pattern (measured in neutron intensity by a $144\times 256$ pixel PSD) and the corresponding SANS model target label. A stack of several images generated with McStas is also shown in Fig. 1. The dataset has been published in Zenodo open access^14^ database, it is intended to be used for Machine Learning purposes, and therefore has been split in train, test, and validation partitions. Each partition is included in a Hierarchical Data Format (HDF) file, which contain two groups: *data* and *target*. The data group is an $n_i\times 144\times 256$ dataset, where $n_i$ is the size of the partition *i*. The target group is an $n_i$ dataset, describing the target label of each array in the partition. Every HDF file comes with a metadata file in comma separated values (csv) format describing the sample and instrument parameters of each virtual experiment in the corresponding partition.

### Recommendation system based on Convolutional Neural Network for SANS

#### Training, testing, and validating on virtual experiments

**Figure 2:**
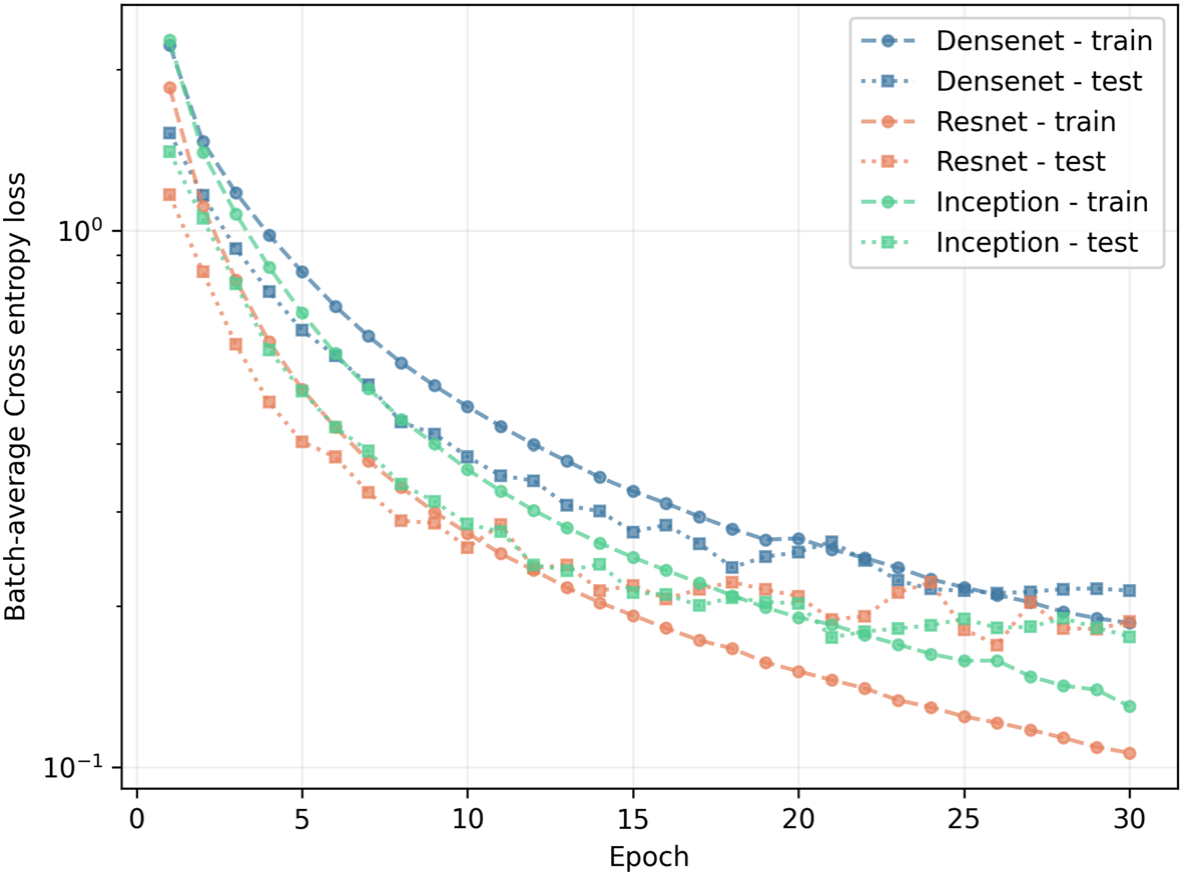
Learning process of the different architectures. Batch-average cross-entropy loss as a function of the number of epochs for all architectures trained in this work, both for training and testing stages of the learning process.

The potential of counting with a dataset of virtual experiments can be seen in this section. We created a SANS model recommendation system based on an ensemble of Convolutional Neural Networks (CNNs). We trained three different architectures of CNN to classify SANS 2D images measured at KWS-1 into a set of 46 SANS models (for more details see the Methods section). By changing the last layer of each CNN to a fully connected layer of size $n_{classes}=46$ and adding a SoftMax layer to the output, we created a predictor for the SANS model label, assigning the predicted label equal to the positional index of the highest weighted node in the SoftMax layer. Then, we used the cross-entropy^15^ loss function to train the neural network and minimize the error of our predictor in the classification task on the SANS model labels. The minimization of the loss function as a function of the epochs (i.e. number of complete passes through the entire training dataset during the training process of our CNNs) may be seen in Fig. 2. Taking into account that the cross entropy loss is shown in logarithmic scale, it takes around 20 epochs for all CNNs to learn the features in the dataset (even earlier in the case of ResNet50). After this point, the minimization steps make the CNNs overfit the training dataset. To avoid overfitting, the accepted weights correspond to the last step in which the batch-average accuracy on the testing dataset decreased with respect to its previous value.

We also created an ensemble model by averaging the SoftMax layer outputs of each of the models trained (see Methods section for more details). Using the validation dataset, we calculated the batch-average accuracy of each CNN and the ensemble model in the classification task and the results can be seen in Table 1. A batch size of 500 was used in this calculation. The Soft-max layer provides us with $c=46$ values (where *c* is the amount of classes in the dataset) between 0 and 1 (adding up to 1), which we try to maximize the value of the correct class by means of the cross entropy loss function. We can rank these values from maximum to minimum, and present the accuracy Top-*k* as the ratio between the number of images in the batch in which the real label is amongst the *k* first values of the SoftMax weights and the total number of images in the batch. Then we average out this value throughout all of the batches. We present the Top-1 (commonly called *accuracy*), Top-3 and Top 5 accuracies. All accuracies were calculated using the 5% of the dataset that was left for validation (validation partition).

**Table 1:** Top-1, Top-3, and Top-5 average accuracies (and corresponding standard deviations) in the classification task of 2D SANS images into SANS models. The accuracy is defined as the ratio between correctly classified images and the total number of images in the partition of the data used. The batch size was 500 and 104 batches were used to calculate the averages and standard deviations.

Model	Top-1 Accuracy	Top-3 Accuracy	Top-5 Accuracy
ResNet50	0.944 ± 0.008	0.994 ± 0.003	0.998 ± 0.001
DenseNet	0.926 ± 0.007	0.988 ± 0.003	0.996 ± 0.002
Inception V3	0.942 ± 0.009	0.994 ± 0.002	0.998 ± 0.001
Ensemble	0.957 ± 0.006	0.996 ± 0.002	0.999 ± 0.001

**Figure 3:**
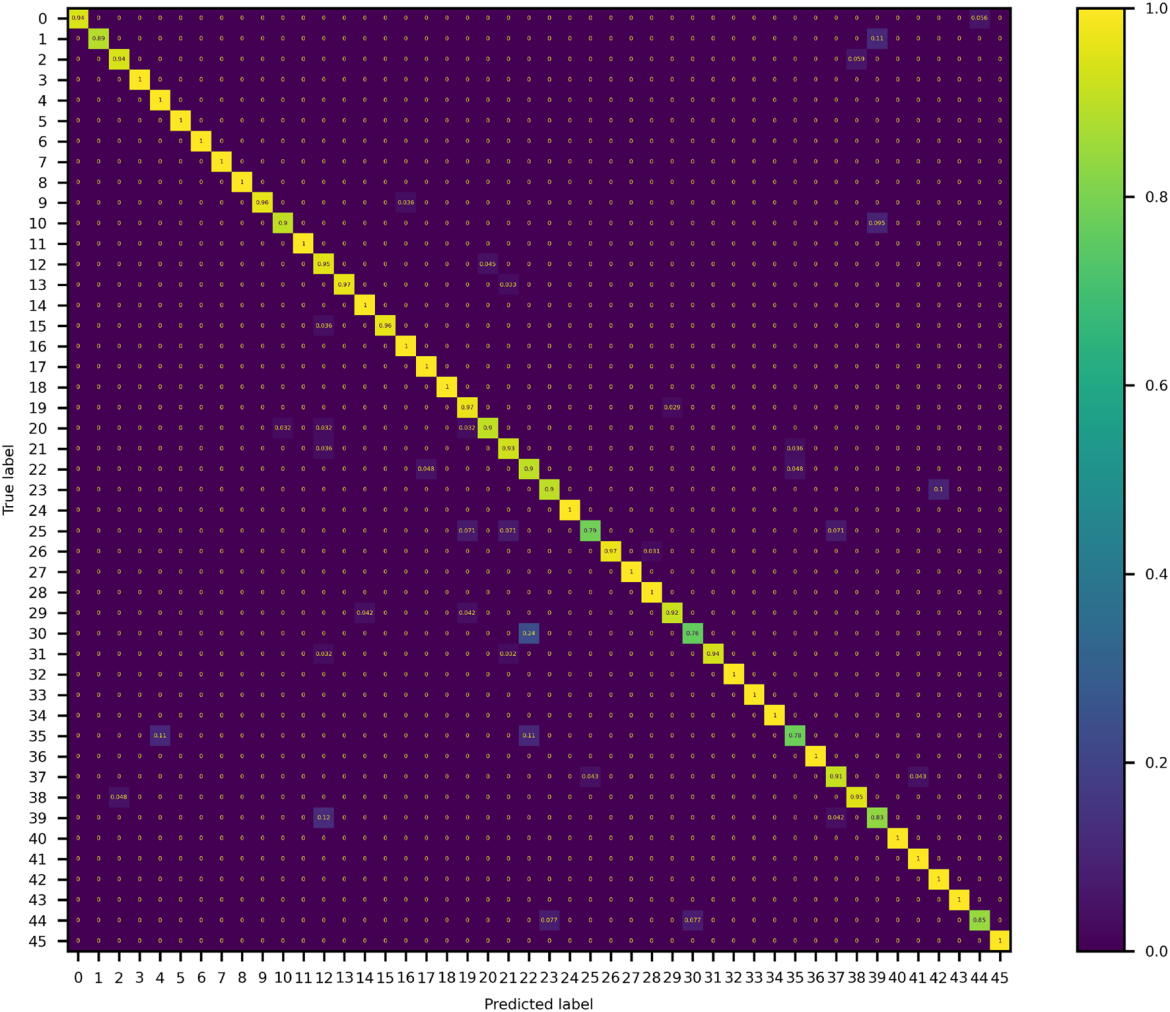
Confusion matrix calculated in the validation set for a batch size of 500 images. The model labels in which there is confusion can be read from Table 2. In the ideal case, a diagonal matrix is desired.

It is possible to see that all three architectures perform similarly, with the ensemble model slightly outperforming the others. Table 1 shows that it is possible to improve the accuracy of our model by generating an ensemble model, which is an observation well studied in other works^16,17^. The main idea of ensemble learning is that by combining multiple models, the errors of a single predictor will likely be compensated by other predictors. This will result in an overall increase in the prediction performance of the ensemble compared to a single predictor. To understand the errors of our trained ensemble model as a recommendation system, we also show the precision, recall and F1 scores^18^ of only those SANS models where there was some confusion (Table 2), as well as the entire confusion matrix of all 46 models (Fig. 3). Some of the models have low scores, but this is correlated with the fact that the dataset is unbalanced. Using stratified sampling, one gets always a smaller support to calculate metrics in less representative classes, such as the *anisotropic Barbell* model (Barbel aniso in Table 2), or the *Polymer micelle* model. This might give a hint to the user into which other models to take into account when a model with high confusion is recommended by the algorithm. There are 15 models that do not show any error in classification in the validation dataset, and these are not shown in Table 2 to focus only on those models which are confused in the classification task.

**Table 2:** Classification report of only those models in which at least one of the metrics was not 1.00 (30 out of 46 models). All other models were classified without any type of error. Anisotropic models (with *aniso* label) are presented first followed by isotropic models. The total support size was 1000, with unbalanced classes, and only one batch was used for this estimation. Precision is defined as the number of True positives correctly predicted over all predicted positives; Recall is the predicted True positives over all real positives; and the F1 score is the harmonic mean of the Precision and Recall. The Label column can be used to identify the models in the confusion matrix in Fig. 3.

Model	Label	Precision	Recall	F1-score	Support
Rectangular prism aniso	37	0.91	0.91	0.91	23
Ellipsoid aniso	15	1.00	0.96	0.98	28
Core shell cylinder aniso	9	1.00	0.96	0.98	28
Bcc paracrystal aniso	2	0.94	0.94	0.94	17
Fcc paracrystal aniso	17	0.95	1.00	0.97	19
Stacked disks aniso	41	0.96	1.00	0.98	25
Cylinder aniso	13	1.00	0.97	0.98	30
Elliptical cylinder aniso	16	0.97	1.00	0.98	28
Core shell ellipsoid aniso	10	0.95	0.90	0.93	21
Sc paracrystal aniso	38	0.95	0.95	0.95	21
Hollow rectangular prism aniso	25	0.92	0.79	0.85	14
Barbell aniso	1	1.00	0.89	0.94	9
Broad peak	4	0.92	1.00	0.96	12
Sphere	39	0.87	0.83	0.85	24
Mass fractal	29	0.96	0.92	0.94	24
Star polymer	42	0.93	1.00	0.96	13
Fractal	19	0.92	0.97	0.94	34
Dab	14	0.97	1.00	0.99	35
Multilayer vesicle	31	1.00	0.94	0.97	31
Lamellar hg	26	1.00	0.97	0.98	32
Adsorbed layer	0	1.00	0.94	0.97	18
Teubner strey	44	0.92	0.85	0.88	13
Polymer micelle	35	0.78	0.78	0.78	9
Lamellar stack paracrystal	28	0.95	1.00	0.98	21
Mono gauss coil	30	0.94	0.76	0.84	21
Fuzzy sphere	21	0.90	0.93	0.91	28
Fractal core shell	20	0.97	0.90	0.93	31
Core shell sphere	12	0.75	0.95	0.84	22
Gel fit	23	0.90	0.90	0.90	10
Gauss lorentz gel	22	0.76	0.90	0.83	21

#### Recommendations with a real measurement

**Figure 4:**
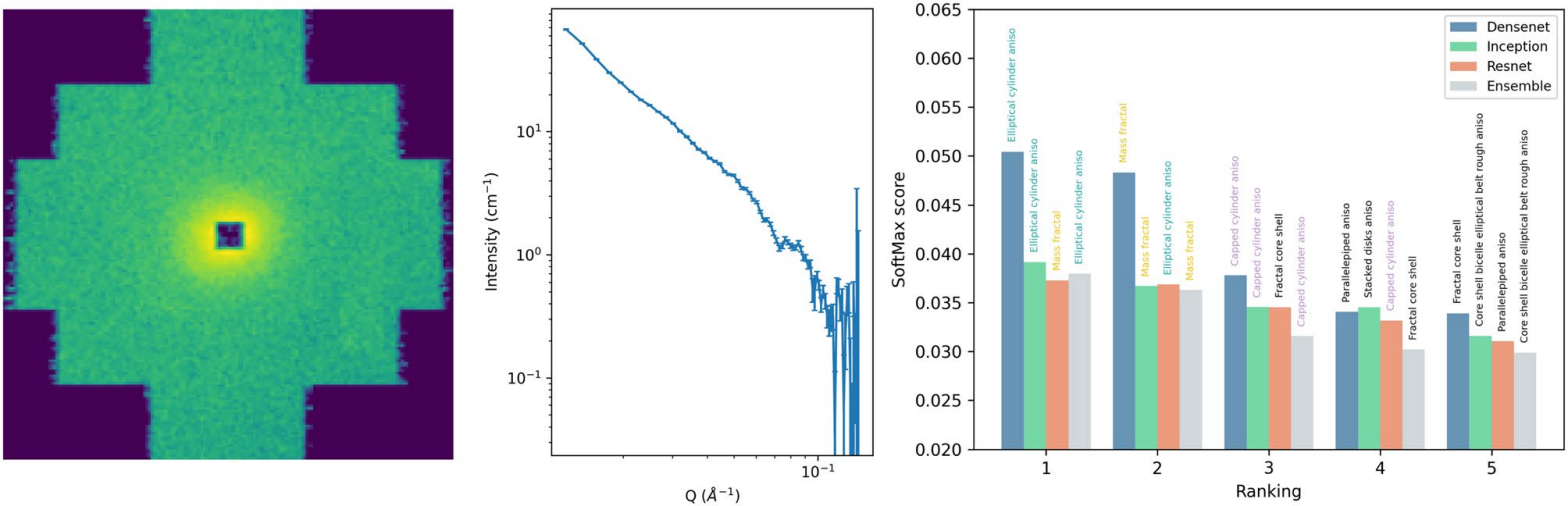
Small angle scattering pattern of a brain-slice sample visualized on the 2D position sensitive detector at the KWS-1 beamline of FRM-II, Garching, Germany (left), the corresponding calibrated I(Q) plot (middle), and the Recommendation plot from our machine learning models (right): five highest scores of the SoftMax layer for each model. Bar color identify the CNN model, and the corresponding recommendation is written on top of each bar. An homogeneous SoftMax vector would have $1/46=0.021$ score value on every score, and is set as the lower baseline of the SoftMax score axis.

To generate discussion about the reach of such an approach to SANS model classification, previously measured and published^19^ experimental data of a mice brain-slice sample was fed to the ensemble, and to each ML model, for SAS model recommendation. The selected 2D image, with its corresponding predictions can be seen in Fig. 4 (see Methods section for more information on the sample). The typical I(Q) representation of the data is also shown in this figure (and can also be found in the corresponding publication with a thorough discussion^19^). This representation is the azimuthal integration result of the 2D image, with an additional transformation from detector pixel position to Q values using the sample to detector distance and the incident neutron wavelength. When integrating the 2D image, information is lost in the process, which may difficult the model selection from 1D curves.

In this example, the form factor would be best modeled with an inner cylinder of relatively large diameter. Then around it, it would be concentric cylinders with alternating scattering length densities. The inner cylinder would have a slightly different scattering length density than the “solvent”, i.e., formalin. As seen in Fig. 4, all of the CNN models trained seem to recommend similar SAS models in the five highest scores of the SoftMax layer. Amongst them, we can find the Elliptical cylinders with anisotropy (i.e. oriented cylinders) and the Mass fractal models, as well as the capped cylinders with anisotropy. These models are highlighted with colors in Fig. 4 and all four networks are recommending them. This suggest that features commonly present in these models and learned in the training are also present in the input data. We leave open the possibility of applying explainable Machine Learning algorithms to obtain further insight on these features.

## Discussion

Monte Carlo simulations play a pivotal role in the advancement of neutron science, particularly in the context of instrument design for large facilities like those used in Small Angle Neutron Scattering experiments. The connection between virtual experiments and Machine Learning has opened up exciting possibilities for fast, accurate, and data-driven optimization of neutron instruments and sample models. The availability of a vast sample description set for SANS models can be intensively exploited for the generation of large virtual experimental datasets to be used in ML applications. The database presented here can be used for teaching purposes, as well as for optimization in instrument design by exploring both, the *sample* and *instrument* parameter descriptions and their corresponding results. An exploratory analysis of this dataset may be fruitful for more insight into the optimization of the KWS-1 beamline.

Although the recommendation system shown here has been trained purely with simulated data, it has been able to detect, identify and discriminate features in SANS images that are inherent to their model description. This local solution of weights that we found, from an optimization point of view, can serve as starting point to accelerate the learning process when a dataset of real experimental data from KWS-1 is present. Like when pre-training a network, using these weights to train a network on a dataset of experimental data of any other instrument can also have good results because the main features, that are characteristics of the form factor models and that we are interested in, have already been extracted. The training in this sense would imply adapting the weights to overcome the experimental contributions of a given instrument, and this could be an interesting approach to model background contributions, which would assist SANS users in this complex task that remains open to optimization.

Once the weights have been adapted to a given beamline under real experimental conditions, this type of trained model can run on the fly while a SANS measurement is taking place. It can start suggesting models during the neutron counting of the detector, and can give an estimate of uncertainty that can then be used as a criteria to stop the measurement and save time. Adding this type of model into neutron and x-ray beamlines is another way in which Machine Learning algorithms can assist users of large-scale experimental facilities.

The real case example of the mouse brain presented here goes to an extreme case in complexity of form factor model description. In these cases, it is natural that none of the trained models fit perfectly the measured data. Despite this, a recommendation system can give insight into which models may be combined to obtain a good fit to the experimental data.

There is also a very important contribution that is not taken into account when performing simulations, that is the background contributions to the measured data, and also the detector imperfections (which can still be, up to a certain point, modelled in a Monte Carlo simulation). Despite this inherent experimental contributions to the measured data, the neural networks can give an estimate and a prediction in these examples too. Even with the “noise” or undesired information of any external contribution, the information that is present in the image shown in Fig. 4 that corresponds to the given form factor model should also be present in the MC dataset if the model (or a similar one) was present during the training phase, and here is where the robustness of the CNNs come into play. The fact that all architectures are recommending the same models amongst the set of 46 models gives us a notion of reliability on the recommendation.

At the moment, we plan to create a dataset of labeled experimental data for the SANS instruments at FRM-II. The necessity of an experimental database similar to the one proposed here is evident in nowadays Machine Learning landscape. This virtual experiment dataset may serve also as an example of what would be desired from a data analyst point of view (formatting of the data, availablity of metadata, etc.) to extract information by means of current ML approaches.

## Methods

### Generation of a dataset of SANS virtual experiments at KWS-1

A code template of the KWS-1 SANS instrument at FRM-II, Garching, was written in McStas (see Supplementary Information for the example code). The instrument description consisted of the following components, set consecutively: a neutron source describing the FRM-II spectrum, a velocity selector, guides that propagate the neutrons to minimize losses, a set of slits to define the divergence of the beam, a sample (one of the recently developed sasmodels component described in the McStas 3.4 documentation), a beamstop, and finally a Position Sensitive Detector (PSD) of size $144\times 256$ pixels. The sample was changed systematically between 46 SAS models (see Supplementary Information for a complete list of the models considered and their documentation), and for each model, different samples were produced by varying the parameters of the model. The set of 46 SAS models considered presented both isotropic and anisotropic scattering amplitudes. In the anisotropic models, the scattering amplitude is defined to have a dependency on the angle between the incident beam and the orientation of the scattering objects (or structures), which is determined by the model parameters. Consequently, in non-oriented particles with analytical anisotropic models, the resulting scattering pattern can result isotropic. Whenever possible, samples were considered in the dilute regime to avoid structure factor contributions and only observe those arising from the form factor. In models with crystalline structure or with correlations between scatterers where an analytical expression for the scattering amplitude was found, the complete scattering amplitude was considered. In all cases, the analytical expressions were obtained from the small angle scattering models documentation of SasView^20^ (see Supplementary Information). The instrument template in the Supplementary Information shows how it was also possible to change the instrument configuration when a sample was fixed. The set of parameters that describe the instrument configuration in a given simulation are referred as *instrument parameters*, and those that define the sample description as *sample parameters*.

In the case of instrument parameters, a discrete set of 36 instrument configurations were allowed to be selected. This was chosen by the instrument scientist, taking into account the most frequent instrument configurations: two possible values of wavelength (4.5 Å  or 6 Å), three possibilities for the distance settings, paired in collimation length - sample to detector distance (8m-1m, 8m-8m, and 20m-20m), three options for the slit configuration (1 cm slit aperture in both directions and a 2 cm wide Hellma Cell; 1.2 cm slit aperture in both directions and a 2cm wide Helma Cell; and 7mm on the horizontal aperture and 1 cm on the vertical aperture with a 1 cm wide Helma Cell), and finally two possible sample holders of different thickness (1mm and 2mm). One of the advantages of MC simulations over analytical approaches to obtain the 2D scattering pattern is that by defining the instrument parameters in the simulation, such as size of apertures for collimation, the sample to detector distance, the size of the detector, the dimensions of the pixels, and so on, the smearing of the data due to instrumental resolution is automatically considered. Therefore, no extra convolution must be performed once the data is collected.

**Figure 5:**
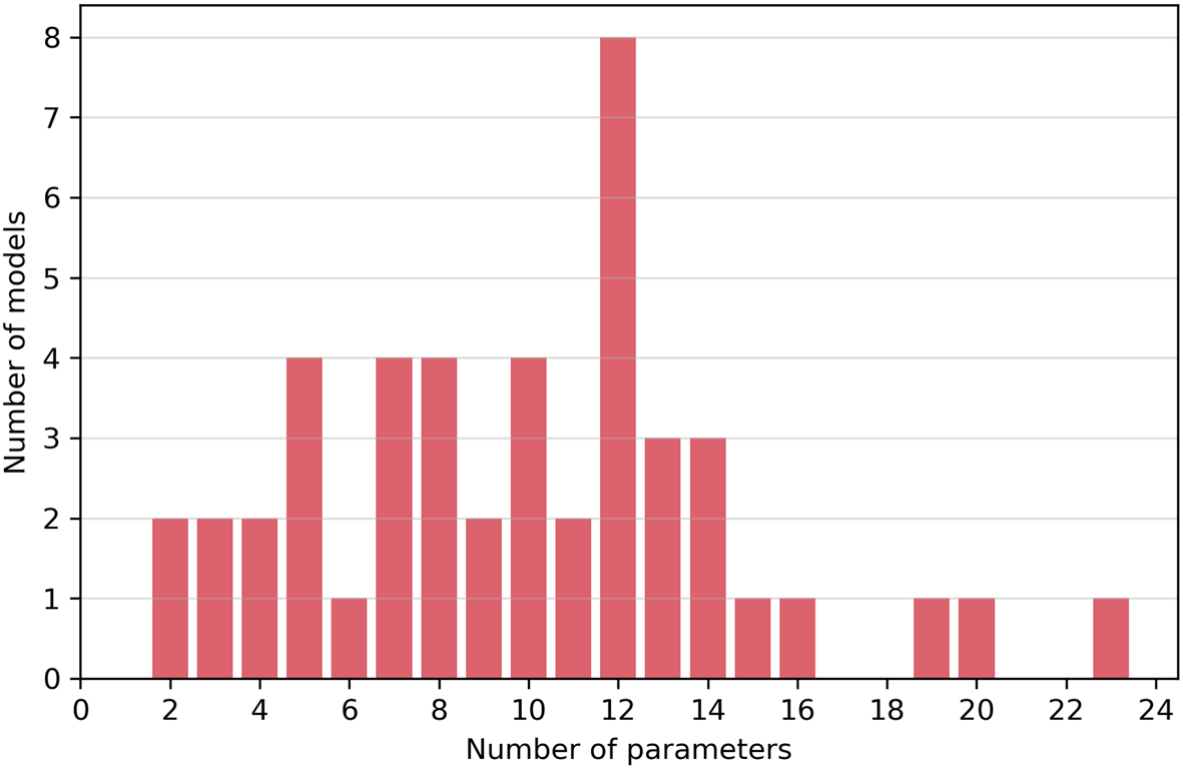
Distribution of models as a function of the number of parameters, showing the wide range of complexities contemplated in the models set used in this work.There are few models that have more than 15 parameters to set.

In the case of sample parameters, most parameters describing samples were continuous, and an added difficulty was that the number of parameters per model was not the same nor similar for all models (see Fig. 5).

There were some models with only two parameters (easy to sample) and several models with more than 15 parameters (hard to sample). Most of the models had around 12 parameters. For *p* parameters with $n_i$ possible choices for parameter *i*, the number of possible combinations (*N*) can be calculated as

1 $$\begin{aligned} N = \prod _{i=1}^p n_i, \end{aligned}$$

which turns out to be $N=n^p$ if $n_i=n$ for all $i=1,\dots ,p$. With only $n=2$ possibilities per parameter and $p=15$, we rapidly get $N=32768$ possible combinations for the complex model, whereas only $N=4$ possible combinations for the very simple models. The large complexity of some model descriptions did not allow simulating all possible scenarios without generating a dataset with a large imbalance between classes. Therefore we opted to sample the defined hyper-parameter space strategically by using latin-hypercube sampling^21^. Briefly explained, this sampling method generates hypercubes in a given high dimensional hyper-parameter space. Then it selects randomly one of these hypercubes, and randomly samples the variables only inside the chosen hypercube. On a later iteration, it selects a new hypercube and repeats the sampling procedure.

Another advantage of MC simulations is that one can perform Monte Carlo integration estimates, which allow to include polydispersity and orientational distributions of scattering objects in a simple and direct manner. On each neutron interaction, the orientation and the polydisperse parameters of the scattering object are randomly chosen from defined probability distributions. For simplicity, distance and dimension parameters $r_i$ of the models were allowed to be polydisperse by sampling them from gaussian distributions (taking care of selecting only positive values). The value $r_i$ selected on each MC simulation defined the mean value of the gaussian distribution and an extra parameter $\Delta r_i$ for each $r_i$ was included in the MC simulation to define the corresponding variance. The standard deviation of the gaussian distribution on different simulations was allowed to vary between 0 (monodisperse) and $r_i/2$ (very polydisperse). In the case of angle parameters that determine the orientation of the scattering object, these were defined by sampling uniformly inside an interval centered at the parameter value $\theta _i$ and with limits defined by another extra parameter $\Delta \theta _i$. For example, in a cylinder form factor model for the scattering object, both the radius and the length of the cylinders can be polydisperse, and the two angles defining the orientation of the principal axis with respect to the incident beam are allowed to vary uniformly within the simulation defined range. This gives a total of 8 parameters to include polidyspersity and orientational distributions on a single simulation. For more information on how this was implemented in the MC simulation we refer the reader to the documentation of each model that is provided in the Supplementary Information.

We opted for sampling 100 points for each sample model in the model’s hyper-parameter space due to time-constraints from the simulation side, and to constraints in the database size from the machine learning side. To define the sampling space, we defined upper ($u_b$) and lower ($l_b$) bounds for each sample parameter in each SasView model description. Then we took the default value of the parameter ($p_{0}$) given in the SasView documentation as the center point of the sampling region, allowing for sampling in the interval $\left[ \max (-3 p_{0},l_b),\min (3 p_{0},u_b)\right]$. All sampled parameters were continuous, except the absorption coefficient, which was restricted to have only two possible values (0% or 10%).

**Figure 6:**
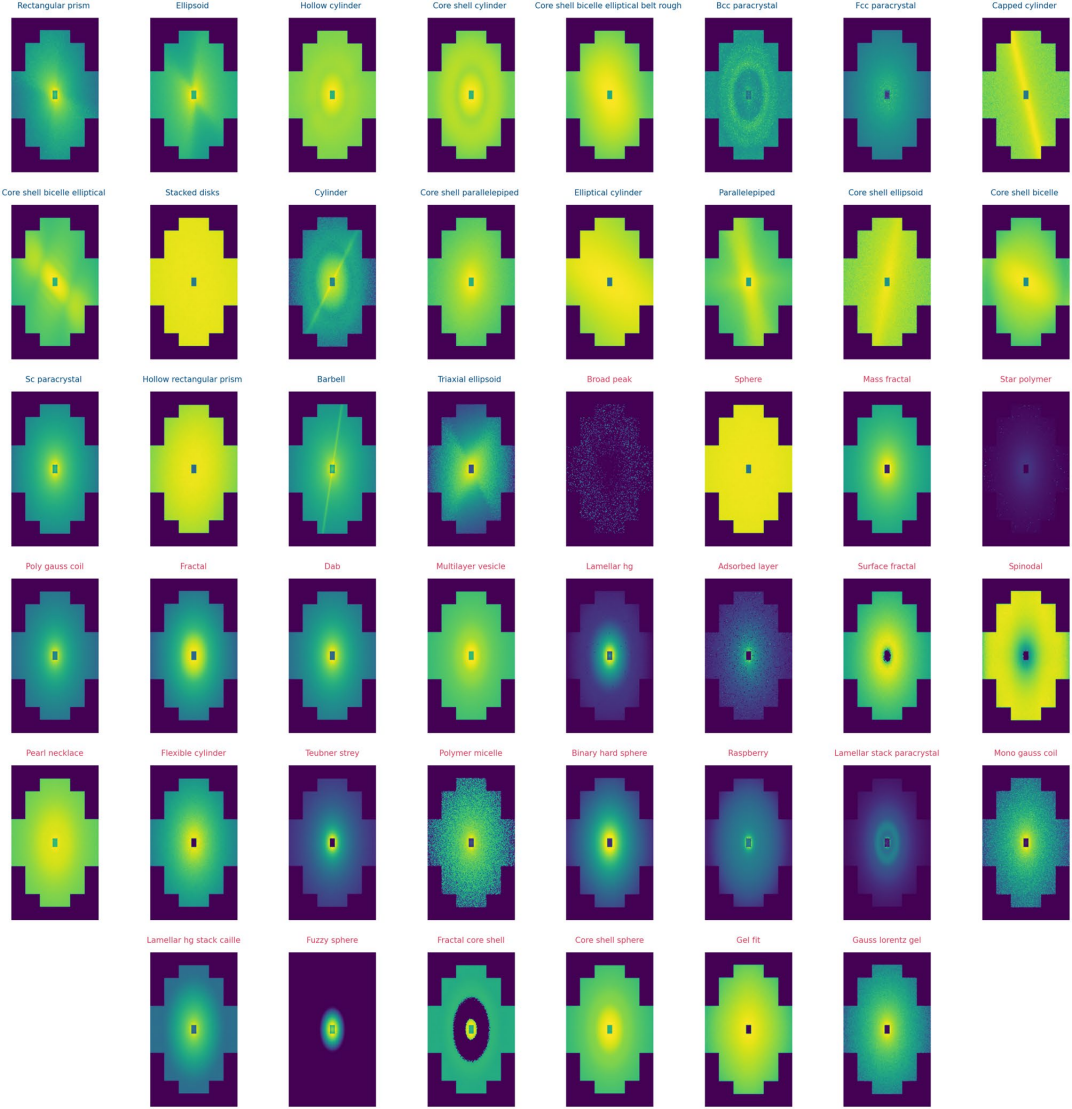
An insight of the variability present amongst models in random images selected from the dataset. Isotropic (red title) and anisotropic (blue title) images can be found, as well as images with high and poor counting statistics.

The expected dataset size was 331.200 by taking the 46 sample models, 2 absorption coefficients, 100 sample parameters per model, and 36 possible instrument settings. The 46 sample models were chosen so as to be representative, and also to avoid those sample models of high computational cost. Given that some configurations were non optimal, the total dataset was cleaned from zero images (no neutrons arrived in the given virtual experiment) and low statistic images. This was executed by calculating the quantile 0.02 of the standard deviations of the images, and removing them from the database. Also, the quantile 0.99 of the maximum value of the pixels of an image was calculated, and all images with max values higher were removed (for example, images in which simulations failed with saturating pixels). A remaining total of 259.328 virtual experiments defined the final dataset for machine learning purposes, and is the dataset published open access^14^. For an insight into what the database looks like we show a random selection of one image per model in the dataset in Fig. 6. It is possible to see that there is some variance between models, but also some unfavorable configurations (inadequate instrument paramaters for a given sample) which add noise and difficulties for the classification task. This figure also illustrates that certain anisotropic SAS models can result in isotropic scattering patterns when the scattering objects are completely unoriented (i.e., exhibiting a broad orientational distribution) or oriented in a particular direction with respect to the beam. In such cases, the anisotropy of the scattering pattern due to the form factor cannot be observed. Consequently, from the perspective of machine learning, the observation of an anisotropic scattering pattern directly excludes all isotropic models, whereas the observation of an isotropic scattering pattern does not allow for the direct inference that the model was isotropic.

### Machine learning approach for SANS model classification

Given that we have a dataset of roughly 260.000 virtual experiments, comprising of a set of 46 SANS models measured under different experimental conditions, we can attempt to train supervised machine learning algorithms to predict the SAS model of a sample given the SANS scattering pattern data measured by the PSD at KWS-1. We are taking advantage here of the fact that we know the ground truth of the SAS model used to generate the data by Monte Carlo simulation. The data from a PSD can be seen as an image of one channel, therefore we can use all recent developments in methods for image classification.

It is known by the SANS community that the intensity profile as a function of the scattering vector (*q*) is normally plotted in logarithmic scale, to be able to see the small features at increasing values of *q*. In this sense, it is useful for the classification task to perform a logarithmic transformation on the measured data to increase the contribution to the image’s variance of the features at large *q*. Since the logarithm is defined only for values larger than 0, and is positive only for values larger than 1, we first add a constant offset of +1 to all pixels and check that there are no negative values in the image. Then we apply the logarithm function to the intensity count in all pixels, emphasizing large *q* features as can be seen in Fig. 6. Then, we normalized all the images in the dataset to their maximum value in order to take them to values between 0 and 1 as to be independent of the counting statistics of the measurement. The transformed data are then fed to the neural network. Mathematically speaking, the transformation reads

2 $$\begin{aligned} x_{i,j} = \frac{\log (x_{i,j}+1.0)}{MaxLog}, \end{aligned}$$

for the intensity of pixel $x_{i,j}$ in row *i* and column *j*, where *MaxLog* is the maximum of the image after applying the logarithmic transformation. All images were resized to $180\times 180$ pixels, since the networks used in this work are designed for square input images. The value 180 is a compromise between 144 and 256, in which we believe the loss in information by interpolation and sampling respectively is minimal. We decided to train Convolutional Neural Networks (CNNs) for the task of classification using Pytorch^22^, by transfering the learning on three architectures (ResNet-50^23^, DenseNet^24^, and Inception V3^25^). In all cases, the corresponding PyTorch default weights were used as starting point and all weights were allowed to be modified. Then, we generated an ensemble method, that averaged the last layer weights of all three CNNs and predicted based on the averaged weight. In all cases, we modified the first layer to accept the generated one-channel images of our SANS database in HDF format. We preferred HDF format to keep floating point precision in each pixels intensity count. Also the final fully-connected layer was modified to match the 46 classes, and a soft-max layer was used to obtain values between 0 and 1, to get some notion of probability of classification.

The dataset was split into training, testing, and validation sets in proportions 0.70, 0.20, and 0.10 respectively. For the minimzation problem in multilabel classification, the Cross Entropy loss is a natural selection as the loss function. This function coincides with the multinomial logistic loss and belongs to a set of loss functions that are called comp-sum losses (loss functions obtained by composition of a concave function, such as logarithm in the case of the logistic loss, with a sum of functions of differences of score, such as the negative exponential)^15^. In our case, we can write the Cross Entropy loss function as

3 $$\begin{aligned} l(x_n,y_n) = -\log \left( \frac{\exp (\alpha _{y_n}(x_n))}{\sum _{c=1}^{C}\exp {(\alpha _{c}(x_n))}}\right) , \end{aligned}$$

where $x_n$ is the input, $y_n$ is the target label, $\alpha _i(x)$ is the *i*-th output value of the last layer when *x* is the input, and *C* is the number of classes. In the extreme case where only the correct weight $\alpha _{y_n}(x_n)$ is equal to 1, the rest are equal to 0, then the quotient is equal to 1, and the logarithm makes the loss function equal to 0. If $\alpha _{y_n}(x_n)<1$, then the quotient will be between 0 and 1, the logarithm will make it negative, and the -1 pre-factor will transform it to a positive value. Any accepted minimization step of this function forces the weight of the correct label to increase in absolute value.

Finally, for the training phase, Mini-batches were used with a batch size of 64 images during training, and all CNNs were trained during 30 epochs. The Adaptive Moment Estimation (Adam)^26^ algorithm was used for the minimzation of the loss function, with a learning rate of $\eta =1\times 10^{-5}$. For the testing phase, a batch size of 500 images was used, and for the validation phase, batches of 1000 images were used to increase the support of the estimated final quantities.

### Data measured at KWS-1

The data was obtained from an already completed study that has been published separetly^19^. It was collected from a sample consisting of a 60 $\mu$m thick brain slice from a reeler mouse after death. In the cited paper^19^, they declare that the animal procedures were approved by the institutional animal welfare committee at the Research Centre Jülich GmbH, Germany, and were in accordance with European Union guidelines for the use and care of laboratory animals. For the interest of this work, we only refer to the data for validation of the presented algorithm and we did not sacrifice nor handle any animal lives. The contrast was obtained by deuterated formalin. The irradiation area was of 1 mm$\times$1 mm. The authors observed an anisotropic Porod scattering ($q<0.04$ Å$^{-1}$) that is connected to the preferred orientation of whole nerve fibres, also called axon. They also report a correlation ring ($q=0.083$ Å$^{-1}$) that arises from the myelin sheaths, a multilayer of lipid bilayers with the myelin basic protein as a spacer.

### Supplementary Information

Supplementary Information 1.

Supplementary Information 2.

## Data Availability

The dataset of the Monte Carlo virtual experiments generated for this work and used here is available at Zenodo open database https://zenodo.org/records/10119316 under a Creative Commons Attribution 4.0 International license.
