# Supplementary material for: Learning from virtual experiments to assist users of Small Angle Neutron Scattering in model selection
Source: Sci Rep. 2024 Jul 1;14:14996. doi: 10.1038/s41598-024-65712-y (PMC11217390; doi:10.1038/s41598-024-65712-y)
Supplement: Supplementary file 1 — Supplementary Information 1. [file 41598_2024_65712_MOESM1_ESM.pdf]

"Learning from virtual experiments to assist users of Small Angle Neutron Scattering in model selection"

Authors: José Ignacio Robledo, Henrich Frielinghaus, Peter Willendrup, and Klaus Lieutenant*
* *j.robledo@fz-juelich.de*

Supplementary Information

Description:
Here we provide the documentation links for all models considered in the Zenodo database "*Small Angle Neutron Scattering (SANS) virtual experiments at KWS-1*" (https://zenodo.org/records/10119316) used to train the recommendation system presented in our work. Both *McStas* and *SasView* links are presented. Source code can be found in McStas documentation, while theoretical descriptions as well as analytical expressions of the models in the SasView documentation.

1. Adsorbed layer: mcstas - https://www.mcstas.org/download/components/3.4_current/sasmodels/SasView_adsorbed_layer.html
sasmodels - https://www.sasview.org/docs/user/models/adsorbed_layer.html
2. Barbell aniso: mcstas - https://www.mcstas.org/download/components/3.4_current/sasmodels/SasView_barbell_aniso.html
sasmodels - https://www.sasview.org/docs/user/models/barbell.html
3. Bcc paracrystal aniso: mcstas - https://www.mcstas.org/download/components/3.4_current/sasmodels/SasView_bcc_paracrystal_aniso.html
sasmodels - https://www.sasview.org/docs/user/models/bcc_paracrystal.html
4. Binary hard sphere: mcstas - https://www.mcstas.org/download/components/3.4_current/sasmodels/SasView_binary_hard_sphere.html
sasmodels - https://www.sasview.org/docs/user/models/binary_hard_sphere.html
5. Broad peak: mcstas - https://www.mcstas.org/download/components/3.4_current/sasmodels/SasView_broad_peak.html
sasmodels - https://www.sasview.org/docs/user/models/broad_peak.html
6. Capped cylinder aniso: mcstas - https://www.mcstas.org/download/components/3.4_current/sasmodels/SasView_capped_cylinder_aniso.html
sasmodels - https://www.sasview.org/docs/user/models/capped_cylinder.html
7. Core shell bicelle aniso: mcstas - https://www.mcstas.org/download/components/3.4_current/sasmodels/SasView_core_shell_bicelle_aniso.html
sasmodels - https://www.sasview.org/docs/user/models/core_shell_bicelle.html
8. Core shell bicelle elliptical aniso: mcstas - https://www.mcstas.org/download/components/3.4_current/sasmodels/SasView_core_shell_bicelle_elliptical_aniso.html
sasmodels - https://www.sasview.org/docs/user/models/core_shell_bicelle_elliptical.html
9. Core shell bicelle elliptical belt rough aniso: mcstas - https://www.mcstas.org/download/components/3.4_current/sasmodels/SasView_core_shell_bicelle_elliptical_belt_rough_aniso.html
sasmodels - https://www.sasview.org/docs/user/models/core_shell_bicelle_elliptical_belt_rough.html
10. Core shell cylinder aniso: mcstas - https://www.mcstas.org/download/components/3.4_current/sasmodels/SasView_core_shell_cylinder_aniso.html
sasmodels - https://www.sasview.org/docs/user/models/core_shell_cylinder.html
11. Core shell ellipsoid aniso: mcstas - https://www.mcstas.org/download/components/3.4_current/sasmodels/SasView_core_shell_ellipsoid_aniso.html
sasmodels - https://www.sasview.org/docs/user/models/core_shell_ellipsoid.html
12. Core shell parallelepiped aniso: mcstas - https://www.mcstas.org/download/components/3.4_current/sasmodels/SasView_core_shell_parallelepiped_aniso.html
sasmodels - https://www.sasview.org/docs/user/models/core_shell_parallelepiped.html
13. Core shell sphere: mcstas - https://www.mcstas.org/download/components/3.4_current/sasmodels/SasView_core_shell_sphere.html
sasmodels - https://www.sasview.org/docs/user/models/core_shell_sphere.html
14. Cylinder aniso: mcstas - https://www.mcstas.org/download/components/3.4_current/sasmodels/SasView_cylinder_aniso.html
sasmodels - https://www.sasview.org/docs/user/models/cylinder.html
15. Dab: mcstas - https://www.mcstas.org/download/components/3.4_current/sasmodels/SasView_dab.html
sasmodels - https://www.sasview.org/docs/user/models/dab.html
16. Ellipsoid aniso: mcstas - https://www.mcstas.org/download/components/3.4_current/sasmodels/SasView_ellipsoid_aniso.html
sasmodels - https://www.sasview.org/docs/user/models/ellipsoid.html
17. Elliptical cylinder aniso: mcstas - https://www.mcstas.org/download/components/3.4_current/sasmodels/SasView_elliptical_cylinder_aniso.html
sasmodels - https://www.sasview.org/docs/user/models/elliptical_cylinder.html
18. Fcc paracrystal aniso: mcstas - https://www.mcstas.org/download/components/3.4_current/sasmodels/SasView_fcc_paracrystal_aniso.html
sasmodels - https://www.sasview.org/docs/user/models/fcc_paracrystal.html
19. Flexible cylinder: mcstas - https://www.mcstas.org/download/components/3.4_current/sasmodels/SasView_flexible_cylinder.html
sasmodels - https://www.sasview.org/docs/user/models/flexible_cylinder.html

20. Fractal: mcstas - https://www.mcstas.org/download/components/3.4_current/sasmodels/SasView_fractal.html
sasmodels - https://www.sasview.org/docs/user/models/fractal.html

21. Fractal core shell: mcstas - https://www.mcstas.org/download/components/3.4_current/sasmodels/SasView_fractal_core_shell.html
sasmodels - https://www.sasview.org/docs/user/models/fractal_core_shell.html

22. Fuzzy sphere: mcstas - https://www.mcstas.org/download/components/3.4_current/sasmodels/SasView_fuzzy_sphere.html
sasmodels - https://www.sasview.org/docs/user/models/fuzzy_sphere.html

23. Gauss lorentz gel: mcstas - https://www.mcstas.org/download/components/3.4_current/sasmodels/SasView_gauss_lorentz_gel.html
sasmodels - https://www.sasview.org/docs/user/models/gauss_lorentz_gel.html

24. Gel fit: mcstas - https://www.mcstas.org/download/components/3.4_current/sasmodels/SasView_gel_fit.html
sasmodels - https://www.sasview.org/docs/user/models/gel_fit.html

25. Hollow cylinder aniso: mcstas - https://www.mcstas.org/download/components/3.4_current/sasmodels/SasView_hollow_cylinder_aniso.html
sasmodels - https://www.sasview.org/docs/user/models/hollow_cylinder.html

26. Hollow rectangular prism aniso: mcstas - https://www.mcstas.org/download/components/3.4_current/sasmodels/SasView_hollow_rectangular_prism_aniso.html
sasmodels - https://www.sasview.org/docs/user/models/hollow_rectangular_prism.html

27. Lamellar hg: mcstas - https://www.mcstas.org/download/components/3.4_current/sasmodels/SasView_lamellar_hg.html
sasmodels - https://www.sasview.org/docs/user/models/lamellar_hg.html

28. Lamellar hg stack caille: mcstas - https://www.mcstas.org/download/components/3.4_current/sasmodels/SasView_lamellar_hg_stack_caille.html
sasmodels - https://www.sasview.org/docs/user/models/lamellar_hg_stack_caille.html

29. Lamellar stack paracrystal: mcstas - https://www.mcstas.org/download/components/3.4_current/sasmodels/SasView_lamellar_stack_paracrystal.html
sasmodels - https://www.sasview.org/docs/user/models/lamellar_stack_paracrystal.html

30. Mass fractal: mcstas - https://www.mcstas.org/download/components/3.4_current/sasmodels/SasView_mass_fractal.html
sasmodels - https://www.sasview.org/docs/user/models/mass_fractal.html

31. Mono gauss coil: mcstas - https://www.mcstas.org/download/components/3.4_current/sasmodels/SasView_mono_gauss_coil.html
sasmodels - https://www.sasview.org/docs/user/models/mono_gauss_coil.html

32. Multilayer vesicle: mcstas - https://www.mcstas.org/download/components/3.4_current/sasmodels/SasView_multilayer_vesicle.html
sasmodels - https://www.sasview.org/docs/user/models/multilayer_vesicle.html

33. Parallelepiped aniso: mcstas - https://www.mcstas.org/download/components/3.4_current/sasmodels/SasView_parallelepiped_aniso.html
sasmodels - https://www.sasview.org/docs/user/models/parallelepiped.html

34. Pearl necklace: mcstas - https://www.mcstas.org/download/components/3.4_current/sasmodels/SasView_pearl_necklace.html
sasmodels - https://www.sasview.org/docs/user/models/pearl_necklace.html

35. Poly gauss coil: mcstas - https://www.mcstas.org/download/components/3.4_current/sasmodels/SasView_poly_gauss_coil.html
sasmodels - https://www.sasview.org/docs/user/models/poly_gauss_coil.html

36. Polymer micelle: mcstas - https://www.mcstas.org/download/components/3.4_current/sasmodels/SasView_polymer_micelle.html
sasmodels - https://www.sasview.org/docs/user/models/polymer_micelle.html

37. Raspberry: mcstas - https://www.mcstas.org/download/components/3.4_current/sasmodels/SasView_raspberry.html
sasmodels - https://www.sasview.org/docs/user/models/raspberry.html

38. Rectangular prism aniso: mcstas - https://www.mcstas.org/download/components/3.4_current/sasmodels/SasView_rectangular_prism_aniso.html
sasmodels - https://www.sasview.org/docs/user/models/rectangular_prism.html

39. Sc paracrystal aniso: mcstas - https://www.mcstas.org/download/components/3.4_current/sasmodels/SasView_sc_paracrystal_aniso.html
sasmodels - https://www.sasview.org/docs/user/models/sc_paracrystal.html

40. Sphere: mcstas - https://www.mcstas.org/download/components/3.4_current/sasmodels/SasView_sphere.html
sasmodels - https://www.sasview.org/docs/user/models/sphere.html

41. Spinodal: mcstas - https://www.mcstas.org/download/components/3.4_current/sasmodels/SasView_spinodal.html
sasmodels - https://www.sasview.org/docs/user/models/spinodal.html

42. Stacked disks aniso: mcstas - https://www.mcstas.org/download/components/3.4_current/sasmodels/SasView_stacked_disks_aniso.html
sasmodels - https://www.sasview.org/docs/user/models/stacked_disks.html

43. Star polymer: mcstas - https://www.mcstas.org/download/components/3.4_current/sasmodels/SasView_star_polymer.html
sasmodels - https://www.sasview.org/docs/user/models/star_polymer.html

44. Surface fractal: mcstas - https://www.mcstas.org/download/components/3.4_current/sasmodels/SasView_surface_fractal.html
sasmodels - https://www.sasview.org/docs/user/models/surface_fractal.html

45. Teubner strey: mcstas - https://www.mcstas.org/download/components/3.4_current/sasmodels/SasView_teubner_strey.html
sasmodels - https://www.sasview.org/docs/user/models/teubner_strey.html

46. Triaxial ellipsoid aniso: mcstas - https://www.mcstas.org/download/components/3.4_current/sasmodels/SasView_triaxial_ellipsoid_aniso.html
sasmodels - https://www.sasview.org/docs/user/models/triaxial_ellipsoid.html
